# Quality of Life and Female Sexual Dysfunction in Croatian Women with Stress-, Urgency- and Mixed Urinary Incontinence: Results of a Cross-Sectional Study

**DOI:** 10.3390/medicina55060240

**Published:** 2019-06-03

**Authors:** Ivan Radoja, Dunja Degmečić

**Affiliations:** 1 Department of Urology, University Hospital Centre Osijek, Faculty of medicine, The J. J. Strossmayer University of Osijek, 31000 Osijek, Croatia; ivan.radoja@yahoo.com; 2 Faculty of medicine, The J. J. Strossmayer University of Osijek, Psychiatric Clinic, University Hospital Centre Osijek, 31000 Osijek, Croatia

**Keywords:** urology, quality of life, female sexual dysfunction, urinary incontinence, women, Croatia

## Abstract

*Background and objectives:* Urinary incontinence is defined as the involuntary leakage of urine. Studies have reported that the severity of urinary incontinence symptoms can cause decreased quality of life and female sexual dysfunction in women, but the association between the duration of the incontinence and the aforementioned disturbances has not been evaluated. The objective of this study was to evaluate the differences in the occurrence of decreased quality of life and female sexual dysfunction in Croatian women with urinary incontinence, with regard to the duration and subtype of urinary incontinence. *Materials and Methods:* We conducted a cross-sectional study from March 2017 to July 2018 at our neurourology and urodynamics outpatient clinic, among 120 women with urinary incontinence symptoms. Based on medical history, physical exam and urodynamic assessment, participants were divided into groups with stress-, urgency- and mixed urinary incontinence. Several quality of life and female sexual dysfunction questionnaires were used for evaluation. The differences between the three UI groups were tested by the Kruskal–Wallis test. All *p* values were two-sided. The level of significance was set to Alpha = 0.05. *Results:* The mixed urinary incontinence group had a significantly inferior quality of life (*p* = 0.003) and lower scores on the female sexual dysfunction questionnaires (*p* = 0.02). The longer the duration of incontinence King’s Health Questionnaire total score was worse (*p* = 0.003) and Female Sexual Function Index total score was worse (*p* < 0.001). *Conclusions:* Our results showed that there was a statistically significant difference in the occurrence of decreased quality of life and female sexual dysfunction considering the duration and subtype of incontinence in Croatian women.

## 1. Introduction

Urinary incontinence (UI) is a common and significant health problem in women, with considerable social, physical, mental and economic impacts on individuals and society, which can cause decreased quality of life (QOL) and female sexual dysfunction (FSD) [1,2]. The relationship between UI, QOL and FSD in women can be difficult to interpret considering the complexity of those issues. Previous original scientific studies found it important to investigate the connection between the severity and type of UI, decreased QOL and the occurrence of FSD, with the help of various specialized questionnaires. First of all, this investigation was conducted to address the problem of prejudice about UI and FSD which are still characterized as a taboo, and secondly to determine an effective, patient-friendly diagnostic evaluation and treatment of UI and FSD. This was done in order to help improve the QOL and sexual function (SF) of patients.

The prevalence of UI in women in developed Western countries ranges between 8% and 55%, and the incidence ranges between 10% and 58%, depending on the age and the will of women to talk about UI [3,4]. There are currently no data on the prevalence and incidence of UI in women in Croatia.

The 2010 International Continence Society (ICS) and International Urogynecological Association (IUGA) joint report defined UI as the complaint of any involuntary leakage of urine [5]. ICS classified UI as the storage lower urinary tract symptom (LUTS) [6,7]. ICS has also defined UI as a condition in which the involuntary leakage of urine is objectively demonstrable and is a social and hygienic problem [8]. Also, the 2010 ICS and IUGA joint report defined the three most common types of UI [5]. Stress urinary incontinence (SUI) was characterized as a complaint of involuntary loss of urine on effort or physical exertion (e.g., sporting activities), or on sneezing or coughing [5]. Urgency urinary incontinence (UUI) was defined as a complaint of involuntary loss of urine associated with urgency [5]. Mixed urinary incontinence (MUI) was described as a complaint of involuntary loss of urine associated with urgency and also with effort or physical exertion, or on sneezing or coughing [5].

The academic and scientific community recommend the World Health Organisation (WHO, Avenue Appia 20, 1211, Geneva 27, Switzerland) definition of QOL [9]. Many scientific studies reported that UI is in most cases a chronic problem, often underreported, left unevaluated and poorly managed. Because of this, affected women can have trouble with interaction among family members, restrictions in physical activities, trouble with sleeping, problems at work, and all this can cause a negative impact on their QOL [10]. In addition, UI is also a considerable financial burden because of high medical treatment costs and the costs of incontinence aids (e.g., pads, diapers), which can also indirectly lead to QOL decline, because we have to consider that some people have a lower socioeconomic status and do not have enough money to purchase such products over a long period, given the chronicity of the UI [11,12].

As far as sexual function (SF) disturbance is concerned, the 2010 ICS and IUGA joint report defined “symptoms of FSD” as a departure from normal sensation and/or function experienced by a woman during sexual activity [5]. FSD is a multicausal medical problem, and the current view regarding the etiology of FSD is best described using the biopsychosocial approach that simultaneously considers physical, psychological, sociocultural, and interpersonal predisposing factors [13]. There are four types of FSD in the Diagnostic and Statistical Manual of Mental Disorders, Fifth Edition (DSM-5): Female Orgasmic Disorder, Female Sexual Interest/Arousal Disorder (formerly known as female disorders of desire and arousal), Genito-Pelvic Pain/Penetration Disorder (formerly known as dyspareunia and vaginismus), and Substance/Medication-Induced Sexual Dysfunction [14]. Hypoactive sexual desire disorder (HSDD) has been eliminated as a diagnostic category in the DSM-5, although the International Society for the Study of Women’s Sexual Health (ISSWSH) terminology still recognizes HSDD as a specific diagnostic entity, consistent with the International Statistical Classification of Diseases and Related Health Problems, 10th Revision (ICD-10) nomenclature system [15]. The anatomical structures of the female reproductive and urinary systems are closely related, so UI can cause FSD in women [16]. The relationship between UI and FSD is apparently logical but it is not entirely clarified. Several studies have investigated the relationship between UI and FSD [17,18].

To our knowledge, no studies have been carried out to determine the effect of the duration of each subtype of UI on FSD and QOL, which could be influential for the enhancement of future larger and higher quality studies on similar topics. The objective of this study was to evaluate the differences in the occurrence and severity of decreased QOL and FSD in Croatian women with UI, with regard to the duration and subtype of UI.

## 2. Materials and Methods

### 2.1. Participants

We conducted a cross-sectional study of the occurrence and severity of decreased QOL and FSD among women with UI in Osijek, Croatia. The study was carried out at the neurourology and urodynamics outpatient clinic of the Urology Department at University Hospital Center Osijek. The study was in compliance with the World Medical Association Declaration of Helsinki. The ethics committee of the University Hospital Center Osijek accepted the survey protocol and approved this study (number of protocol: R2:13417-5/2016; 16 September 2016). Women who visited our outpatient clinic because of their UI symptoms were asked to participate in the study. The patients were enrolled in the research after reading and signing informed consent. The inclusion criteria for our research were women with one of the aforementioned three subtypes of UI, age above 18 years and interest in participating in the research. The exclusion criteria were women aged under 18 years and those who either had active urinary infection, urolithiasis, urothelial cancer or gynecological cancer. We managed to collect the required sample of the three UI subtype groups (SUI, UUI and MUI groups, each consisting of 40 women) in the period from March 2017 to July 2018. There were 14 women with UI examined in the aforementioned period who were unwilling or unable to participate in our survey.

### 2.2. Procedure

To determine the type of UI, all women were assessed by neurourological physical exam, medical history and urodynamic techniques and measurements in accordance to the ICS standards. The assessment also included several QOL and FSD questionnaires, which have been used in similar previous original scientific studies. To evaluate QOL, FSD and the presence, severity and intensity of UI symptoms in a good manner, all women filled out the questionnaires alone in a safe and friendly environment in a separate, isolated room, after the physical examination and urodynamics. It was necessary to allocate approximately 30 min of time to complete the questionnaires. All the questions were formulated in a simple and understandable way, and women answered sincerely about QOL and SF.

### 2.3. Questionnaires

Patients who agreed to participate in the study received the following questionnaires during their examination in order to collect the necessary data for research: sociodemographic questionnaire, 3-day bladder diary, International Consultation on Incontinence Questionnaire—short form (ICIQ-UI-SF) [19], WHO QOL questionnaire—short version (WHOQOL-BREF) [20], King’s Health Questionnaire (KHQ) [21], Pelvic Organ Prolapse/Incontinence Sexual Questionnaire, IUGA-Revised (PISQ-IR) [22] and Female Sexual Function Index (FSFI) [23]. All of the questionnaires were well-established methods that were used in previous similar studies to evaluate QOL and FSD, and they all had scoring and interpretation instructions. To establish good content validity for the Croatian version, the questionnaires were translated into the Croatian language from the English version by a colleague with a Bachelor of Arts in English Language and Literature degree by the Department of English, Faculty of Humanities and Social Sciences, The J. J. Strossmayer University of Osijek.

We used a standardized sociodemographic questionnaire which included data about age, duration of UI symptoms, sexual orientation, place of residence, living conditions, marital status, employment status, use of medications, cigarettes and alcohol, and number of births.

The frequency of micturition, the volume of single voided volume, urgency, the situation in which the UI episode occurred, the number of urine “leakage” episodes, and the volume of urine that involuntarily “leaked” were estimated through a 3-day bladder diary.

QOL was measured using three questionnaires: ICIQ-UI-SF, WHOQOL-BREF and KHQ. ICIQ-UI-SF provided us with insight into the frequency and volume of involuntary urine leakage, UI severity and impact on QOL, and information on situations in which incontinence occurred. We have divided the ICIQ-UI-SF score into four severity levels [24]. We first divided the ICIQ-UI-SF score into four levels with the QOL dimension among the three UI groups: slight (1–5), moderate (6–12), severe (13–18) and very severe (19–21). Then we divided the ICIQ-UI-SF score into four severity levels without a QOL dimension among the three UI groups: slight (1–3), moderate (4–5), severe (6–9) and very severe (10–11). WHOQOL-BREF produced a more detailed QOL profile. The first two questions asked about an individual’s overall perception of QOL and about an individual’s overall perception of their health. After that, the individual’s perception of QOL was denoted by questions associated with four domains: psychological health, level of independence, social relations and environment. KHQ also gave us an extensive understanding of QOL through nine domains. The first part of the questionnaire had questions about general health perception and incontinence impact, while the second part addressed daily activity limitations, physical and social limitations, personal relationships, emotions, quality of sleep, daily energy and severity measures.

FSD was measured using two questionnaires: PISQ-IR, in which we had a special insight into the SF problems of sexually inactive and sexually active women, and FSFI, with the help of which we gained insight into FSD considering the normal sexual response cycle in women. FSFI was used to assess different aspects of SF in the period over the last 4 weeks before the women answered the questionnaire in our outpatient clinic. Each domain had components that were specifically scored and then multiplied by a certain coefficient, and a total score that was ≤26.55 was classified as FSD, as stated in the FSFI scoring instructions.

### 2.4. Statistics

To discern the mean effect of the numeric variables of the three independent groups, with the significance level of 0.05, power 0.8, and the effect size 0.29, the minimum sample size required was 111 subjects (37 subjects per group) (calculated by G * Power 3.1.2, Franz Faul, University of Kiel, Germany) [25]. Regarding the statistical analysis of the results, categorical data were represented by absolute and relative frequencies. Numerical data were described by median and interquartile range, in case of deviation from the normal distribution. The variance of the category variables was tested by the Chi-square test and Fisher’s exact test. The differences between variables in two independent groups were tested by the Mann–Whitney U Test. The differences between three UI groups were tested by the Kruskal–Wallis test. The correlation between numeric variables was evaluated by Spearman’s correlation coefficient ρ (rho). All P values were two-sided. The level of significance was set to Alpha = 0.05. The statistical analysis was conducted using the MedCalc Statistical Software version 18.2.1 and the International Business Machines Corporation (IBM) Statistical Package for the Social Sciences (SPSS) statistical software, version 23 [26,27].

## 3. Results

### 3.1. Sociodemographic Questionnaire and 3-Day Bladder Diary

The overall analyzed sample was 120 women with UI, divided into three groups of 40 participants, according to the three most common subtypes of UI: SUI, UUI and MUI groups. Regarding sexual orientation, all women declared themselves heterosexual. The duration of UI in the overall analyzed sample measured in years ranged from 1 to 30 years, with a median of 5 years interquartile range (IQR) from 3 to 9 years, and this was not significantly different among the observed UI groups. The sociodemographic characteristics of participants are presented in Table 1 and Table 2.

Results obtained from the 3-day bladder diary are presented in Table 3 and Table 4. The number of urgency episodes that happened over a 24-hour period was higher in women who had UUI and lower in women with SUI. The number of pads used per day, the episodes of incontinence and the approximate amount of involuntary urine leakage that happened over a 24-hour period was not significantly different among the observed UI groups.

### 3.2. Quality of Life

The ICIQ-UI-SF results of situations in which UI occurred are presented in Table 5. In the MUI group, more episodes of UI occurred while going to the toilet and for no apparent reason. The ICIQ-UI-SF score with the four severity levels with QOL dimension is presented in Table 6. The ICIQ-UI-SF score with the four severity levels without QOL dimension is presented in Table 7. The longer the duration of UI, the participants rated their QOL as significantly worse (Table 8).

The WHOQOL-BREF results are presented in Table 9. The longer the symptoms of UI were present, the participants had considerably lower total WHOQOL-BREF scores, lower psychological health, social relations and environment scores.

The KHQ grade scale was from 0 (full satisfaction with QOL) to 1127 points (totally dissatisfied with QOL). The participants with MUI had a worse overall KHQ result in comparison with SUI or UUI participants, and a lower result in the KHQ incontinence impact domain and KHQ physical limitations domain compared to the other two UI groups (Table 10).

The longer the symptoms of UI were present, the participants had a considerably lower overall perception of health. Also, we have established that the greater the incontinence impact, the poorer the QOL, and this showed the strongest correlation with the duration of UI. The total KHQ score, the scores of general health perception, incontinence impact domain, role limitations, physical limitations and emotions were lower with a longer duration of UI (Table 11).

### 3.3. Female Sexual Dysfunction

PISQ-IR gave us insight into the FSD in sexually inactive and active women. Of the total number of participants, 69 were sexually active (only 15 participants in the MUI group). Of the total number of participants, 87 had a sexual partner, without significant differences between the UI groups. Of those 87 participants who had a sexual partner, there were 69 sexually active women and 18 sexually inactive women (Table 12). Distribution by sexual activity and the presence of a sexual partner is presented in the Table 13.

Spearman’s coefficient of correlation was used to evaluate the correlation between FSD and the duration of UI in sexually inactive participants. In all sexually inactive subjects, there was a significant positive correlation between the duration of UI and partner-related SF domain (Table 14).

Also, Spearman’s rank correlation coefficient was used to evaluate the correlation between SF and the duration of UI in sexually active participants. There was a significant negative correlation between the duration of UI and the excitement/orgasm domain, and between the duration of UI and partner-related domain (Table 15).

FSFI gave us an insight into the SF of participants during the last 4 weeks before answering the questionnaire, through six domains. The score of FSFI was significantly lower in participants with MUI. The participants with SUI and UUI had similar FSFI scores. The desire domain was better scored in the group with UUI. The satisfaction domain was better scored in the group with SUI. There were no significant differences regarding the orgasm, lubrication and pain domains in all three UI groups, although the scores were lower. The Kruskal–Wallis test results of the six FSFI domains and total FSFI score of all three UI groups with regard to sexual activity are presented in Table 16 and Table 17. We noticed that a longer duration of UI is associated with a lower FSFI score (Table 18).

## 4. Discussion

The longer the duration of UI, the participants rated their QOL significantly worse. Total KHQ score and the general perception of health had lower results. UI had a greater general impact on QOL, daily and physical limitations and emotional status. The participants with MUI were significantly more dissatisfied with QOL, they had significantly lower total KHQ scores due to general impact of UI on QOL, and more physical limitations compared to the other two groups. Other studies also showed that women with MUI had lower QOL than women with SUI and UUI [28]. Participants also had lower results regarding their psychological health, social relations, environment and the total WHOQOL-BREF scale result.

The FSFI score was lower in women with MUI and higher in the SUI and UUI groups. The results for the SUI group were very similar to previous scientific studies, maybe because SUI is more predictable, therefore, women may have used coping strategies more often (e.g., voiding before coitus) [29,30]. We were surprised to find that the total FSFI score was similar in the SUI and UUI groups and so different compared to the MUI group, in which we thought that the urgency component was the more dominant one with regard to the questions about situations in which UI occurred inside the ICIQ-UI-SF. Involuntary urine leakage during penetration is present in the majority of cases in women with SUI, probably because of the modification of the anatomical position of the bladder neck and the urethra during the friction of the erect penis inside the vagina [31]. Involuntary urine leakage during orgasm is mostly present in women with UUI, probably because orgasm can provoke hyperactivity of the urinary bladder detrusor muscle [31]. The results of FSFI orgasm domain were similar in both groups but the domain questions did not refer to incontinence. In all the questionnaires that we used in our study there was no direct question as to whether involuntary leakage of urine occurred during an orgasm, during penetration or throughout the intercourse. We can only speculate what was the real reason behind these results. These questions about coital urinary incontinence should be addressed in an adequate way inside a more specific questionnaire in future studies.

We have noticed that a longer duration of UI is associated with a lower overall FSFI score. This result may be related to the genital complaints of women. Chronic involuntary urine leakage can cause skin and mucosa inflammation of the vulva and vagina, which can lead to genito-pelvic pain/penetration disorder and other female sexual disorders [32].

FSFI domains of sexual desire and pain during intercourse had the lowest scores among all participants. Participants reported decreased sexual pleasure, which over time could decrease arousal and lubrication and cause pain, which could further decrease pleasure and desire, regarding the current state of research in the field of FSD.

Given the obtained results and the objective of our study, we have shown that there is a difference in the occurrence and severity of decreased QOL and FSD with regard to the duration and subtype of UI. There were some clinical implications and confounding factors regarding the results of the FSD questionnaires in the sexually active and sexually inactive women. All sexually inactive women with a longer duration of involuntary urine leakage episodes had higher partner-related domain scores according to PISQ-IR, which indicated a greater impact of the duration of UI on their SF associated with avoiding any kind of sexual activity with a sexual partner, or avoiding finding a sexual partner. In our study we did not want to focus on the methods of treating UI and FSD. We wanted to emphasize that if physicians could openly communicate with women about their sexual inactivity related to their UI symptoms and use specialized FSD questionnaires, maybe they could encourage them to become sexually active again, find a sexual partner, use behavioral treatment for sexual problems, permission, limited information, specific suggestions, and intensive therapy (PLISSIT) model, or advise them to consult a certified specialist in sexual medicine [33]. In all the three UI groups, all sexually active women had a sexual partner and they had significantly better FSFI scores, although it was <26.55 and defined as FSD. Therefore, we can assume that the presence of the marital or sexual partner and the practice of penile-vaginal sexual intercourse has a positive effect on all parts of the sexual response cycle in women, including physical and emotional intimacy, vaginal lubrication and pelvic floor function. According to the data available on scientific and biomedical databases, so far no one has estimated the impact of the duration of UI on the occurrence and severity of FSD and decreased QOL, but the observations were related to the subtype of UI and the severity of the UI symptoms [34,35]. Moreover, previous original scientific studies did not simultaneously use all of the questionnaires utilized in our research [36,37]. A recent review article has reported, according to a database analysis, that KHQ was individually used in six studies (42.86%), ICIQ-UI-SF in four studies (28.57%) and FSFI in two studies (14.28%), and only some articles used more than one questionnaire [38]. The problematic components of our research were the fact that the research was conducted only in our neurourology and urodynamics clinic in University Hospital Center Osijek during a relatively short period of time, and a relatively small number of women participated in the research. This was the source of selection bias and our results cannot be generalized to the overall population in Croatia.

Since we do not have data on the number of women with UI in Osijek and in Croatia, our sample was probably inadequately represented and therefore maybe this was also a source of selection bias. The participants with MUI had similarities regarding the fact that they had both UUI and SUI symptoms, so this was a potential source of sampling bias. Regarding non-response bias, there were 14 women with UI examined in the aforementioned period that were unwilling or unable to participate in our survey.

Women are generally reluctant to talk about problems related to involuntary urine leakage because they are ashamed, uninformed of treatment options and convinced that UI is a natural, inevitable and integral part of ageing. It was difficult to gather the required sample for our research because in Croatia incontinence and sexuality are still controversial themes and women were at first reluctant to participate in our research. As far as other sources of potential bias were concerned, we did not take into account or investigate the influence of other aspects on the onset of FSD, for instance estrogen status in women, the health of their sexual partner and frequent or habitual partner change among participants.

## 5. Conclusions

The issues associated with UI are very challenging and they require a very detailed analysis, which can be facilitated with the help of various specialized questionnaires. The identification, prevalence, incidence and relationship between UI, QOL and FSD described in previous scientific studies were dependent on whether women with UI sought medical attention and whether they agreed to participate in the research. So, there is relatively small number of original scientific studies about this topic and a small number of respondents in those studies, which makes it difficult to assess the state of the problem of the overall population.

There are different interpretations about what is the normal and abnormal degree of involuntary leakage of urine within different cultural, social, age and sex groups, and its degree of influence on the QOL of the individual. Depending on social circumstances and subjective expectations, an individual’s reaction to involuntary urine leakage can change from one situation to another, which can lead to a deterioration in the QOL to a greater or lesser extent. Very mild leakage of urine can have a major and devastating impact on one individual, and on the other hand, somebody with more severe leakage may manage it with less significance.

Because UI is usually a chronic problem, a longer duration of UI can have a major impact on QOL and FSD. Women with UI worry about urine odor and the loss of urine during coitus, so they often avoid sexual contact and consequently the frequency of sexual intercourse can decrease which can cause FSD. FSD can cause problems within marriages and sexual partner-related problems, which can have strong influence on the occurrence of decreased QOL. Decreased QOL is one of the psychosocial consequences in women with UI, despite the increasing availability of safe and effective types of treatment for UI.

It is necessary to raise awareness in women about the benefits of prompt recognition and discussion about UI issues and related FSD with a physician, in order to improve QOL. It is also necessary to inform physicians about the possibility of using questionnaires during the evaluation, not only of UI but of other LUTS. Future studies should take into account the duration, severity and subtype of UI, assess other risk factors that can lead to the onset of FSD, include more participants over a longer period of time in different outpatient clinics, and describe the results achieved by using multiple QOL and FSD screening questionnaires. Also, it is important to include in the future studies questions about involuntary urine leakage with regard to penetration and female sexual response cycle models. This could lead to an early recognition of the aforementioned disturbances associated with UI in women, minimize possible complications and negative consequences, because of the prompt treatment based on the individual approach.

## Figures and Tables

**Table 1:** Sociodemographic characteristics of participants.

Urinary Incontinence (UI) Group	Median (Interquartile Range)				*p* *
	Stress Urinary Incontinence (SUI) (*n* = 40)	Urgency Urinary Incontinence (UUI) (*n* = 40)	Mixed Urinary Incontinence (MUI) (*n* = 40)	Total (*n* = 120)	
Age (years)	55 (49–63)	55 (47–61)	61 (53–67)	57 (49–64)	0.03
Duration of UI (years)	5 (3–10)	4.5 (2–7)	5 (3–10)	5 (3–9)	0.24
Number of childbirths	2 (2–2)	2 (1–2)	2 (1–2)	2 (1–2)	0.19

* Kruskal–Wallis test.

**Table 2:** Sociodemographic characteristics of participants.

	Number (%) of Participants				*p* *
	SUI	UUI	MUI	Total	
Marital status					
Married	27 (67.5)	28 (70)	22 (55)	77 (64.2)	0.72
Cohabitation	1 (2.5)	1 (2.5)	3 (7.5)	5 (4.2)	
Single	10 (25)	8 (20)	10 (25)	28 (23.3)	
Divorced	2 (5)	3 (7.5)	5 (12.5)	10 (8.3)	
Total	40 (100)	40 (100)	40 (100)	120 (100)	
Population of residence					
<1000	4 (10)	3 (7.5)	12 (30)	19 (15.8)	0.03 ^†^
1001–10,000	5 (12.5)	7 (17.5)	7 (17.5)	19 (15.8)	
10,001–50,000	11 (27.5)	5 (12.5)	5 (12.5)	21 (17.5)	
>50,000	20 (50)	25 (62.5)	16 (40)	61 (50.8)	
Total	40 (100)	40 (100)	40 (100)	120 (100)	
Level of education					
Elementary school	12 (37.5)	6 (18.8)	16 (41)	34 (33)	0.11 ^†^
High school	15 (46.9)	15 (46.9)	18 (46.2)	48 (46.6)	
College	5 (15.6)	11 (34.4)	5 (12.8)	21 (20.4)	
Total	32 (100)	32 (100)	39 (100)	103 (100)	
Employment status					
Employed	13 (32.5)	22 (55)	10 (25)	45 (37.5)	0.03
Unemployed	12 (30)	9 (22.5)	10 (25)	31 (25.8)	
Occasionally employed	0	1 (2.5)	0	1 (0.8)	
Retired	15 (37.5)	8 (20)	20 (50)	43 (35.8)	
Total	40 (100)	40 (100)	40 (100)	120 (100)	

* Fisher’s exact test; ^†^ χ^2^ test.

**Table 3:** 3-day Bladder Diary results.

	Median (Interquartile Range)				*p* *
	SUI (*n* = 40)	UUI (*n* = 40)	MUI (*n* = 40)	Total (*n* = 120)	
Episodes of urgency per day	3 (2–7)	6 (5–9)	5 (3–9)	6 (4–8)	0.01
Episodes of UI per day	5 (3–7)	5 (4–7)	6 (4–10)	5 (4–7)	0.06
Number of pads per day	2 (2–4)	2 (2–3)	3 (2–4)	2 (2–4)	0.26

* Kruskal–Wallis test.

**Table 4:** 3-day Bladder Diary results.

	Number (%) of Participants				*p* *
	SUI	UUI	MUI	Total	
Approximate amount of involuntary leakage of urine					
Small	19 (47.5)	19 (47.5)	14 (35)	52 (43.3)	0.38
Medium	15 (37.5)	18 (45)	17 (42,5)	50 (41.7)	
Large	6 (15)	3 (7.5)	9 (22,5)	18 (15)	
Total	40 (100)	40 (100)	40 (100)	120 (100)	

*χ^2^ test.

**Table 5:** Situations in which involutary urine leakage occurred in relation to UI groups.

	Number (%) of Participants				*p* *
	SUI (*n* = 40)	UUI (*n* = 40)	MUI (*n* = 40)	Total (*n* = 120)	
Before going to the toilet	3 (7.5)	23 (57.5)	23 (57.5)	49 (40.8)	<0.001
Coughing and sneezing	38 (95)	2 (5)	34 (85)	74 (61.7)	<0.001
Sleeping period	0	2 (5)	11 (27.5)	13 (10.8)	<0.001
Physical activity	35 (87.5)	0	16 (40)	51 (42.5)	<0.001
After urination	4 (10)	10 (25)	10 (25)	24 (20)	0.14
No obvious reason	5 (12.5)	33 (82.5)	29 (72.5)	67 (55.8)	<0.001
All the time	2 (5)	2 (5)	2 (5)	6 (5)	>0.99

* χ^2^ test.

**Table 6:** International Consultation on Incontinence Questionnaire—short form (ICIQ-UI-SF) Score with the quality of life (QOL) Dimension.

	Number of Participants (%)				*p* *
	SUI (*n* = 40)	UUI (*n* = 40)	MUI (*n* = 40)	Total (*n* = 120)	
Slight (1–5)	3 (8)	0	1 (3)	4 (3.3)	0.08
Moderate (6–12)	4 (10)	11 (28)	6 (15)	21 (17.5)	
Severe (13–18)	29 (73)	27 (68)	25 (63)	81 (67.5)	
Very severe (19–21)	4 (10)	2 (5)	8 (20)	14 (11.7)	
Total	40 (100)	40 (100)	40 (100)	120 (100)	

* Fisher’s exact test.

**Table 7:** ICIQ-UI-SF Score without the QOL Dimension.

	Number of Participants (%)				*p* *
	SUI (*n* = 40)	UUI (*n* = 40)	MUI (*n* = 40)	Total (*n* = 120)	
Slight (1–3)	2 (5)	0	2 (5)	4 (3.3)	0.24
Moderate (4–5)	1 (3)	4 (10)	2 (5)	7 (5.8)	
Severe (6–9)	35 (88)	33 (83)	29 (73)	97 (80.8)	
Very severe (10–11)	2 (5)	3 (7.5)	7 (17.5)	12 (10)	
Total	40 (100)	40 (100)	40 (100)	120 (100)	

* Fisher’s exact test.

**Table 8:** The association of QOL with duration of UI according to ICIQ-UI-SF.

	Spearman’s Rank Correlation Coefficient	
	Rho (95% CI)	*p* Value
Overall QOL considering duration of UI	0.283 (0.08 to 0.41)	0.002

**Table 9:** The association of QOL with duration of UI according to WHO QOL questionnaire—short version (WHOQOL-BREF).

	Spearman’s Rank Correlation Coefficient	
	Rho (95% CI)	*p* Value
Physical health	−0.143 (−0.31 to 0.04)	0.12
Psychological health	−0.339 (−0.49 to −0.17)	<0.001
Social relations	−0.345 (−0.49 to −0.18)	<0.001
Environment	−0.301 (−0.46 to −0.13)	0.001
Total WHOQOL-BREF score	−0.358 (−0.51 to −0.20)	<0.001

**Table 10:** The association of KHQ results with type of UI according to KHQ.

	Median (Interquartile Range)				*p* *
	SUI (*n* = 40)	UUI (*n* = 40)	MUI (*n* = 40)	Total (*n* = 120)	
General health perception	50 (50–75)	50 (25–50)	50 (50–75)	50 (50–50)	0.12
Incontinence impact	66.6 (66.6–66.6)	66.6 (66.6–100)	100 (66.6–100)	66.6 (66.6–100)	0.003
Role limitations	50 (33.3–50)	50 (33.3–66.6)	66.6 (33.3–100)	50 (33.3–66.6)	0.08
Physical limitations	50 (33.3–66.6)	50 (33.3–66.6)	83.3 (66.6–100)	66.6 (33.3–83.3)	<0.001
Social limitations	33.3 (22.2–61.1)	44.4 (33.3–66.6)	44.4 (33.3–88.8)	44.4 (30.5–66.6)	0.12
Personal relationships	33.3 (33.3–66.6)	50 (33.3–66.6)	66.6 (33.3–100)	50 (33.3–66.6)	0.05
Emotions	44.4 (22.2–66.6)	33.3 (33.3–52.7)	55.5 (22.2–86)	44.4 (27.8–66.6)	0.36
Sleep/energy	33.3 (16.6–50)	33.3 (33.3–50)	50 (33.3–66.6)	33.3 (33.3–50)	0.07
Severity measures	66.6 (58.3–83.3)	66.6 (66.6–75)	75 (60.4–91.6)	75 (58.3–83.3)	0.11
Total King’s Health Questionnaire (KHQ) score	272.4 (817.4–424.7)	377.7 (802.2–427.9)	379.6 (868–533.2)	445.9 (342.2–602.1)	0.02

* Kruskal–Wallis test.

**Table 11:** The association of KHQ results with the duration of UI.

	Spearman’s Rank Correlation Coefficient	
	Rho	*p* Value
General health perception	0.289	0.002
Incontinence impact	0.375	<0.001
Role limitations	0.241	0.01
Physical limitations	0.189	0.04
Social limitations	0.123	0.21
Personal relationships	0.051	0.66
Emotions	0.279	0.003
Sleep/energy	0.101	0.31
Severity measures	0.100	0.28
Total KHQ score	0.270	0.003

**Table 12:** Sexually active participants with a sexual partner in relation to UI groups.

	Number (%) of Participants				*p* *
	SUI	UUI	MUI	Total	
Sexually active	25 (62.5)	29 (72.5)	15 (37.5)	69 (57.5)	0.005
W/sexual partner	30 (75)	33 (82.5)	24 (60)	87 (72.5)	0.07

* χ^2^ test.

**Table 13:** Distribution by sexual activity and the presence of a sexual partner.

	Number (%) of Participants			*p* *
	Sexually Inactive	Sexually Active	Total	
W/O sexual partner	33 (64.7)	0	33 (27.5)	<0.001
W/sexual partner	18 (35.3)	69 (100)	87 (72.5)	
Total	51 (100)	69 (100)	120 (100)	

* Fisher’s exact test.

**Table 14:** The correlation between FSD and the duration of UI in sexually inactive participants.

	Spearman’s Rank Correlation Coefficient	
	Rho	*p* Value
All sexually inactive participants		
Partner-related	0.325	0.02
Condition specific	0.083	0.59
Global quality rating	0.014	0.92
Condition impact	0.021	0.89
Total Pelvic Organ Prolapse/Incontinence Sexual Questionnaire, IUGA-Revised (PISQ-IR) score	0.053	0.70
All sexually inactive participants without sexual partner		
Partner-related	0.240	0.18
Condition specific	−0.107	0.59
Global quality rating	−0.045	0.81
Condition impact	−0.069	0.73
Total PISQ-IR score	−0.269	0.13
All sexually inactive participants with sexual partner		
Partner-related	0.295	0.29
Condition specific	0.211	0.47
Global quality rating	0.264	0.32
Condition impact	0.330	0.23
Total PISQ-IR score	0.327	0.19

**Table 15:** The correlation between female sexual disfunction (FSD) and the duration of UI in sexually active participants.

	Spearman’s Rank Correlation Coefficient	
	Rho	*p* Value
Arousal, orgasm	−0.282	0.02
Partner-related	−0.406	0.001
Condition specific	0.012	0.93
Global quality rating	−0.039	0.75
Condition impact	−0.170	0.17
Desire	−0.062	0.61
Total PISQ-IR score	−0.168	0.17

**Table 16:** The results of Female Sexual Function Index (FSFI) questionnaire in UI groups regardless of sexual activity.

	Median (Interquartile Range)				*p* *
	SUI	UUI	MUI	Total	
Desire	3 (1.2–4.2)	3.6 (2–4.2)	1.2 (1.2–3.5)	3 (1.2–3.6)	0.004
Arousal	4.2 (3.6–5.1)	3.7 5 (3.3–4.2)	3.9 (2.4–4.5)	3.9 (3.3–4.7)	0.10
Lubrication	4.2 (3–4.8)	3.6 (3–4.2)	3.8 (3.1–4.6)	3.6 (3–4.5)	0.70
Orgasm	4.4 (3.6–4.8)	3.6 (3.23–4)	4.0 (3.1–4.4)	4.0 (3.3–4.4)	0.12
Satisfaction	3.8 (1.8–4.4)	3.6 (2.4–4.4)	0.8 (0.8–4)	3.2 (0.8–4.4)	0.03
Pain	4.4 (3.8–4.8)	3.6 (2.8–4.2)	3.6 (2.4–4.5)	3.6 (3.2–4.8)	0.02
Total FSFI score	19.9 (2–27)	19.8 (4.3–24.1)	2.0 (1.7–19.3)	17.1 (2–23.4)	0.02

* Kruskal–Wallis test.

**Table 17:** Evaluation of total FSFI score in relation to sexual activity and UI groups.

	Median (Interquartile Range)				*p* *
	Sexually Active W/Partner	Sexually Inactive W/Partner	Sexually Inactive W/O Partner	Total	
SUI total FSFI score	24.4 (20.8–28.7)	2 (1.4–11.1)	1.4 (1.2–2.2)	19.9 (2–27)	<0.001
UUI total FSFI score	22.2 (19.1–24.8)	3.3 (2.2–4.9)	1.2 (1.2–2.0)	19.8 (4.3–24.1)	<0.001
MUI total FSFI score	23.2 (17.1–25.1)	2.0 (1.9–4.0)	1.8 (1.2–2.0)	2.0 (1.7–19.3)	<0.001

* Kruskal–Wallis test.

**Table 18:** The association of total FSFI score and the duration of UI.

	Spearman’s Rank Correlation Coefficient	
	Rho (95% CI)	*p* Value
Total FSFI score	−0.376 (−0.52 to −0.21)	<0.001
